# Integrated approach for studying bioactive compounds from *Cladosporium* spp. against estrogen receptor alpha as breast cancer drug target

**DOI:** 10.1038/s41598-022-22038-x

**Published:** 2022-12-27

**Authors:** Satish Anandan, Hittanahallikoppal Gajendramurthy Gowtham, C. S. Shivakumara, Anjana Thampy, Sudarshana Brijesh Singh, Mahadevamurthy Murali, Chandan Shivamallu, Sushma Pradeep, Natarajamurthy Shilpa, Ali A. Shati, Mohammad Y. Alfaifi, Serag Eldin I. Elbehairi, Joaquín Ortega-Castro, Juan Frau, Norma Flores-Holguín, Shiva Prasad Kollur, Daniel Glossman-Mitnik

**Affiliations:** 1 Department of Clinical Nutrition and Dietetics, Sri Devaraj Urs Academy of Higher Education and Research, Kolar, Karnataka 563101 India; 2 Department of PG Studies in Biotechnology, Government Science College (Autonomous), Nrupathunga Road, Bangalore, 560001 India; 3 Department of Studies in Botany, University of Mysore, Manasagangotri, Mysore, Karnataka 570006 India; 4 Department of Biotechnology and Bioinformatics, JSS Academy of Higher Education and Research, Mysore, Karnataka 570015 India; 5 Department of Studies in Microbiology, University of Mysore, Manasagangotri, Mysore, Karnataka 570006 India; 6 Biology Department, Faculty of Sciences, King Khalid University, Abha, Saudi Arabia; 7 Cell Culture Lab, Egyptian Organization for Biological Products and Vaccines (VACSERA Holding Company), 51 Wezaret El-Zeera St., Agouza, Giza, Egypt; 8 Departament de Química, Universitat de les Illes Balears, 07122 Palma de Mallorca, Spain; 9 Laboratorio Virtual NANOCOSMOS, Departamento de Medio Ambiente y Energía, Centro de Investigación en Materiales Avanzados, 31136 Chihuahua, Chih Mexico; 10 School of Physical Sciences, Amrita Vishwa Vidyapeetham, Mysuru Campus, Mysuru, Karnataka 570026 India

**Keywords:** Cheminformatics, Medicinal chemistry, Theoretical chemistry, Biochemistry, Cancer, Chemical biology, Computational biology and bioinformatics, Drug discovery, Chemistry

## Abstract

*Cladosporium* spp. have been reported for their great diversity of secondary metabolites which represent as a prominent base material for verifying the biological activities. Several bioactive compounds which have antimicrobial, cytotoxic, quorum sensing inhibitory and phytotoxic activities have been isolated from *Cladosporium* species. Most of them are still needed to be explored for their anticancer properties. Therefore, the present study is focused on screening and identifying the bioactive compounds of *Cladosporium* spp. for their anticancer activity via the integrated approaches of Molecular Docking (MD), Molecular Dynamics Simulation (MDS) and Density Functional Theory (DFT) studies. A total of 123 bioactive compounds of *Cladosporium* spp. were explored for their binding affinity with the selected breast cancer drug target receptor such as estrogen receptor alpha (PDB:6CBZ). The Molecular Docking studies revealed that amongst the bioactive compounds screened, Altertoxin X and Cladosporol H showed a good binding affinity of − 10.5 kcal/mol and − 10.3 kcal/mol, respectively, with the estrogen receptor alpha when compared to the reference compound (17$\upbeta$-Estradiol: − 10.2 kcal/mol). The MDS study indicated the stable binding patterns and conformation of the estrogen receptor alpha-Altertoxin X complex in a stimulating environment. In addition, in silico absorption, distribution, metabolism, excretion and toxicity (ADMET) study suggested that Altertoxin X has a good oral bioavailability with a high LD$_{50}$ value of 2.375 mol/kg and did not cause any hepatotoxicity and skin sensitization. In summary, the integrated approaches revealed that Altertoxin X possesses a promising anticancer activity and could serve as a new therapeutic drug for breast cancer treatment.

## Introduction

Presently, breast cancer is one of the most common cancers in women in the world in terms of 2.26 million new cases and 0.685 million deaths occurring worldwide in 2020^1,2^. The global breast cancer mortality rate is alarmingly high due to delayed diagnosis or treatment availability^3^. Approximately 80% of breast cancers in women are from hormone-dependent estrogen receptor positive breast cancer. Since the estrogen receptor alpha is mainly responsible for the initiation and progression of breast cancer, it has emerged as the single most important target for treating the disease^4,5^. Therefore, there is a lot of emphasis on developing new effective therapeutic drugs which specifically bind to estrogen receptor alpha and prevent the progression of hormone-dependent breast cancer. To date, many ligands such as estradiol, genistein, daidzein, biochanin A, 6,3’,4’-trihydroxyflavone, phloretin, ellagic acid, (-)-epigallocatechin-3-gallate, ursolic acid, kaempferol, naringenin, toxaphene, chlordane, thiadiazole acrylamide (XCT790), diethyl stilbestrol and deketene curcumin derivatives have been successfully identified as antagonists of estrogen receptor alpha by the virtual screening of structural model4^4,6,7^. Even though plants have long been utilized to treat several diseases, including cancer, the bioactive compounds produced by microbes have yet to be investigated. Although plants have long been utilized to treat several diseases, including cancer, the bioactive compounds produced by microbes have yet to be investigated.

The availability of many microbial-originated antibiotics in the market^8^ has gained a great interest in the discovery of anticancer drug lead compounds from the unexplored microbial sources, especially fungi. Fungi are well-known for their ubiquitous occurrence as they are one of the most abundant sources of chemodiversity^9,10^. The genus *Cladosporium* is considered as a rich source of diverse and bioactive natural compounds that are reported for their biological properties including antimicrobial, cytotoxic, quorum sensing inhibitory and phytotoxic activities belonging to various classes of secondary metabolites such as naphtalenones, alkaloids, flavonoids, lactones, benzofluoranthenones, macrolides, coumarins, perylenequinones, azaphilones, isocoumarins, sterols and others^11^. Taxol extracted from *Cladosporium oxysporum* has been extensively reported for its anticancer as well as antibacterial properties^12^. However, most of the compounds in *Cladosporium* spp. have not yet been exploited for their anticancer properties.

The use of bioinformatics tools to find drugs that could be used to combat several diseases is commonplace nowadays. Structure-based drug design enables virtual screening of compounds for possible therapeutic candidates by analyzing their binding affinities with protein receptors before in vitro and in vivo experiments^13,14^. The integrated computational approaches of MD, MDS and DFT calculation are used to analyze their performance during interaction with protein receptors. Therefore, the present study was focused on the exploration of *Cladosporium* spp. bioactive compounds for their anticancer properties through the integrated approaches of Molecular Docking, Molecular Dynamics Simulation and DFT studies as well as absorption, distribution, metabolism, excretion and toxicity (ADMET) prediction calculations. The estrogen receptor alpha, a key transcription factor in breast cancer was used as the breast cancer drug target.

## Materials and methods

### Optimization of ligands

A total of 123 bioactive compounds from *Cladosporium* spp. reported from a recently published literature^11^ and the reference compound (17$\upbeta$-estradiol) were selected for molecular docking and simulation studies against the estrogen receptor alpha (PDB: 6CBZ) protein receptor responsible for breast cancer. Since the estrogen receptor alpha is an important biological target mediating 17$\upbeta$-estradiol driven breast cancer development, 17$\upbeta$-estradiol was used as the reference compound in the study. The 3D and 2D structures of the compounds were downloaded from the online PubChem database (https://pubchem.ncbi.nlm.nih.gov/) in structure data file (SDF) format. Some of them were created using MarvinSketch (version 18.30) ChemAxon chemical drawing tool (https://chemaxon.com/products/marvin/). The 2D structures were converted into 3D coordinates and geometries which are then converted into protein data bank (PDB) format using the open-source chemical toolbox, Open Babel^15^. Before molecular docking, the geometries of the ligand PDB files were optimized in the PRODRG server (https://davapc1.bioch.dundee.ac.uk/cgi-bin/prodrg/submit.html). These optimized structures were used as the ligand molecules for the molecular docking studies.

### Preparation and validation of the protein receptor

The three-dimensional structure of the estrogen receptor alpha ligand-binding domain Y537S mutant in complex with estradiol and GRIP peptide (PDB: 6CBZ) was retrieved from the PDB database (https://www.rcsb.org/) in PDB format. The protein receptor was prepared for Molecular Docking by removing ligand and water molecules attached to it using Discovery Studio Visualizer (version 20.1.0.19295) software to avoid interfering with the docking study. The energy minimization studies were performed with empirical force fields using Swiss-Pdb Viewer software (version 4.10) to generate the protein structure’s lower energy conformations, which infers a greater stable conformation. The process optimizes conformational errors in the structure’s geometry during the protein structure modeling. In addition, the steepest descent algorithm was employed using the GROMOS 96 force field for the geometry optimization process^16^. The PDBsum database (https://www.ebi.ac.uk/thornton-srv/databases/pdbsum/Generate.html) was used to validate the protein structure with the Ramachandran plot^17^, which suggested that the majority (96.8%) of the amino acids residues were found within the most favored regions of the protein used.

### Molecular docking study

The AutoDock Vina implicated in PyRx Virtual Screening software (version 0.8) was used to execute the molecular docking study by considering the estrogen receptor alpha (PDB: 6CBZ) protein and bioactive compounds as macromolecule and ligand molecules, respectively. AutoDock Vina was used for fixing the cubical grid box size at 60 $\times$ 60 $\times$ 60 with 0.375 Å around the active sites of the protein. The 100 independent docking runs were performed for each compound. The most favorable binding pose was selected based on the lowest free binding energy (kcal/mol). The molecular interaction between the amino acid residues of protein receptors and ligand molecules was studied by visualizing the docking result using Discovery Studio Visualizer software. The accuracy of the docking protocol was validated through re-docking (self-docking) of the compounds with the protein used during the study.

### Molecular dynamics simulations study

The molecular dynamics simulations for the protein alone and protein-ligand complex were carried out using the GROMACS software (version 5.1.4) with the AMBER force field, modified Berendsen thermostat and LINear Constraint Solver (LINCS) constraint algorithm. The docked complex was cleaned and optimized first, followed by the orientation of hydrogen bond network systems. The parameter files were generated for the ligand using SwissParam web server. The TIP3P solvation model was used to solvate the cubic simulation system with water and NaCl counter-ions were added to neutralize the charge of the system. The steepest (gradient) descent algorithm was used to perform the initial energy minimization of the system with 5000 steps. The system was then equilibrated using NVT (or canonical) ensemble followed by NPT (isothermal-isobaric) ensemble for 100 ps. The protonation states of key amino acid residues such as Histidine (His) in 6CBZ protein as well as selected ligand molecules (Altertoxin X, Cladosporol H and 17$\upbeta$-estradiol) were analyzed at neutral pH using the PROPKA web server, which provided the pKa value around His residues^18^. Finally, the molecular dynamics simulations were performed for 100 ns with an integration time of 0.002 ps under the physiological parameters (temperature: 300 K and pressure: 1 bar) of simulation systems. The simulation trajectories were exploited to calculate the root mean square deviation (RMSD), root mean square fluctuation (RMSF), the radius of gyration (Rg), solvent accessible surface area (SASA) and the number of hydrogen bonds.

### Binding free energy calculations

The outcome of the MD simulation run for the target protein complexed with Altertoxin X, Cladosporol H and 17$\upbeta$-Estradiol was subjected to binding free energy calculations using the Molecular MechanicsPoisson-Boltzmann Surface Area (MM-PBSA) technique. It was another application of molecular dynamics simulations and thermodynamics for determining the extent of ligand binding with protein. The gmx$\_$MMPBSA program with MMPBSA Stat.py script, which utilizes the GROMACS 2018.1 trajectories as input, was used to determine the binding free energy for each ligand-protein combination. The gmx$\_$MMPBSA program used three components to calculate the binding free energy: molecular mechanical energy, polar and apolar solvation energies, and molecular mechanical energy. The calculations were based on MD trajectories of last 100 ns, which compute $\Delta$G with dt 1000 frames. It was evaluated using molecular mechanical energy, polar and apolar solvation energies. The equations (1) and (2) used to calculate the free binding energy are given below.

1 $$\begin{aligned} \Delta G_{Binding}= & {} \Delta G_{Complex} - (G_{Protein} + G_{Ligand}) \end{aligned}$$

2 $$\begin{aligned} \Delta G= & {} \Delta E_{MM} + \Delta G_{Solvation} - T \Delta S = \Delta E_{(Bonded + Non-bonded)} + \Delta G_{(Polar+ Non-polar)} - T \Delta S \end{aligned}$$

where G$_{Binding}$: Binding free energy, G$_{Complex}$: Total free energy of the protein-ligand complex, G$_{Protein}$ and G$_{Ligand}$: Total free energies of the isolated protein and ligand in solvent, respectively, $\Delta$G: Standard free energy, $\Delta$E$_{MM}$: Average molecular mechanics potential energy in vacuum, $\Delta$G$_{Solvation}$: Solvation energy, $\Delta$E: Total energy of bonded as well as non-bonded interactions, $\Delta$S: Change in entropy of the system upon ligand binding; T. Temperature in Kelvin.

### Conceptual DFT studies

The molecular energy, electronic density, and frontier orbital energies, chemical reactivity descriptors and of the studied Altertoxin X, Cladosporol H and 17$\upbeta$-Estradiol molecular systems were determined using the Kohn-Sham (KS) approach^19,20^ while making use of the Conceptual DFT (CDFT) methodology^21–24^. Many different conformers of the studied compounds were determined using MarvinView 17.15 from ChemAxon [http://www.chemaxon.com] through the consideration of the MMFF94 force field to perform Molecular Mechanics calculations^25–29^. This was followed by a geometry optimization and frequency calculation by means of the Density Functional Tight Binding (DFTBA) methodology^30^ and a later geometry reoptimization, frequency analysis and calculation of the electronic properties and the chemical reactivity descriptors by means of the MN12SX/Def2TZVP/H2O model chemistry^31–33^ on their optimized molecular structures. The charge of the molecule was taken as equal to zero while the radical anion and cation have been considered in the doublet spin state. This determination was performed with the aid of the Gaussian 16 software^30^ and the SMD solvation model^34^ and owing to the fact that the mentioned model chemistry has been previously proved as verifying the ’Koopmans in DFT’ (KID) procedure^35–38^, This last step was also required for the verification of the absence of imaginary frequencies as a check for the stability of the optimized structure as being a minimum in the energy landscape.

### Absorption, distribution, metabolism, excretion and toxicity (ADMET) predictions

The physicochemical and ADMET properties of the selected potential compounds were predicted by utilizing the pkCSM platferm^39^. These parameters are related to the absorption, distribution, metabolism, excretion and toxicity of drugs.

- **Absorption parameters:** water solubility in a buffer system, Caco2 cell permeability, intestinal (human) absorption, P-glycoprotein inhibition and skin permeability.
- **Distribution parameters:** Lipinski’s rule, blood-brain barrier (BBB) and central nervous system (CNS) permeability.
- **Metabolism parameters:** Cytochrome P450 (CYP)1A2, CYP2C19, CYP2C9, CYP2D6 and CYP3A4 inhibition, CYP2D6 and CYP3A4 substrate.
- **Excretion parameters:** total renal clearance and renal OCT2 substrate.

Toxicity parameters: AMES test, oral rat acute and chronic toxicity, skin sensitization, hepatotoxicity, Tetrahymena pyriformis toxicity and Minnow toxicity.

## Results and discussion

Extensive studies have demonstrated the anticancer properties of the compounds found in many plants, but microbial compounds’ use for cancer treatment are still yet to be explored. In the present study, the binding potential of bioactive compounds from *Cladosporium* spp. was explored with the estrogen receptor alpha through the integrated in silico approaches to identify inhibitory effect. The estrogen receptor alpha is a critical transcription factor for human breast cancer development, which accounts for approximately 70% of breast cancer^40^. It significantly contributes to the progression and inhibition of breast cancer in women. Due to its over-expression, the estrogen receptor alpha could help us to find the new effective ways to treat the breast cancer. Therefore, the estrogen receptor alpha was used as a promising protein target to determine the drug candidate for breast cancer therapy.

### Molecular docking studies

**Figure 1:**
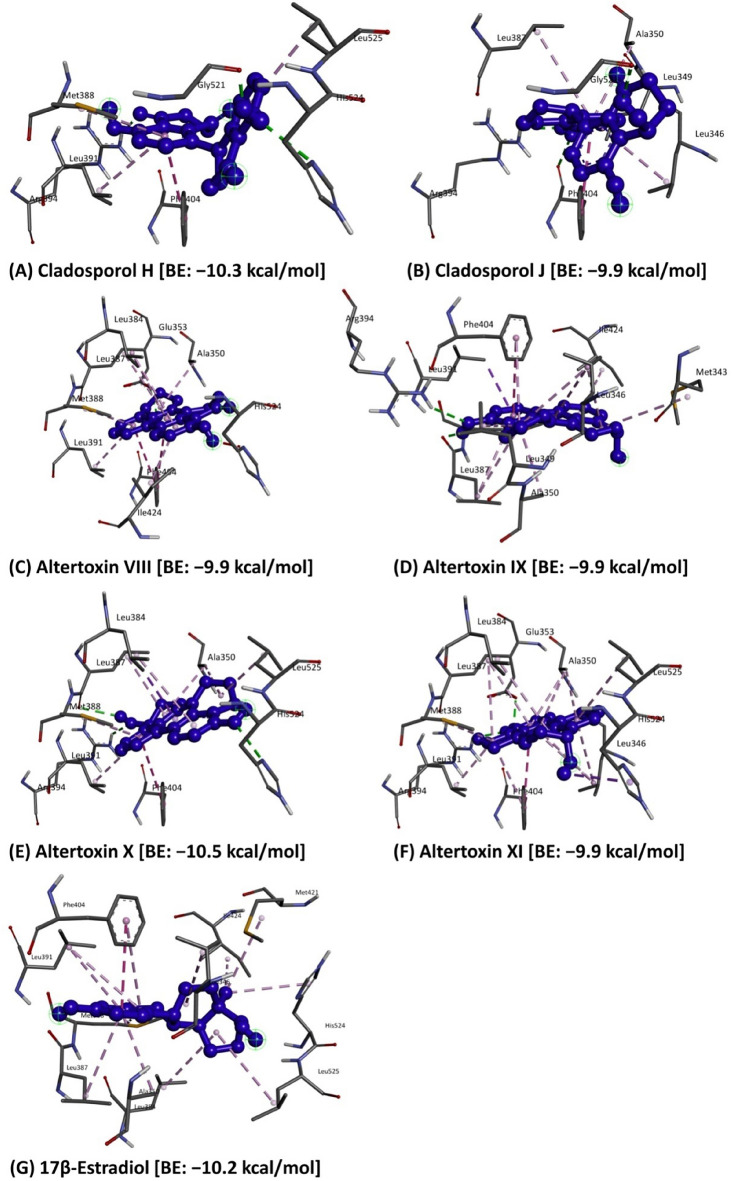
Docking model of the potential bioactive compounds of *Cladosporium* spp. in the active binding site of the estrogen receptor alpha (PDB: 6CBZ) protein.

The outcome of the molecular docking analysis suggested that six bioactive compounds (*viz.*, Cladosporol H, Cladosporol J, Altertoxin VIII, Altertoxin IX, Altertoxin X and Altertoxin XI) were found to obtain the higher binding affinities ranging from − 9.9 to − 10.5 kcal/mol (Table 1). The interaction of compounds with the protein target (PDB: 6CBZ) resulted in varied binding potential. Among the evaluated compounds, the best binding energy results were noticed with Altertoxin X (− 10.5 kcal/mol) followed by Cladosporol H (− 10.3 kcal/mol), while − 10.2 kcal/mol was noticed with the reference compound 17$\upbeta$-Estradiol. These results indicated that the binding affinity was increased by modifying (replacing) the hydroxyl (-OH) group with a long carbon chain/aromatic ring molecule^41^. The best binding pose clearly suggested that the potential bioactive compounds as drugs candidates bind within the estrogen receptor alpha (Fig. 1). The 2D schematics indicated that amino acids played a significant role in the pattern of interactions between the protein and the ligands, significantly contributing to the total energy of the interaction.

**Figure 2:**
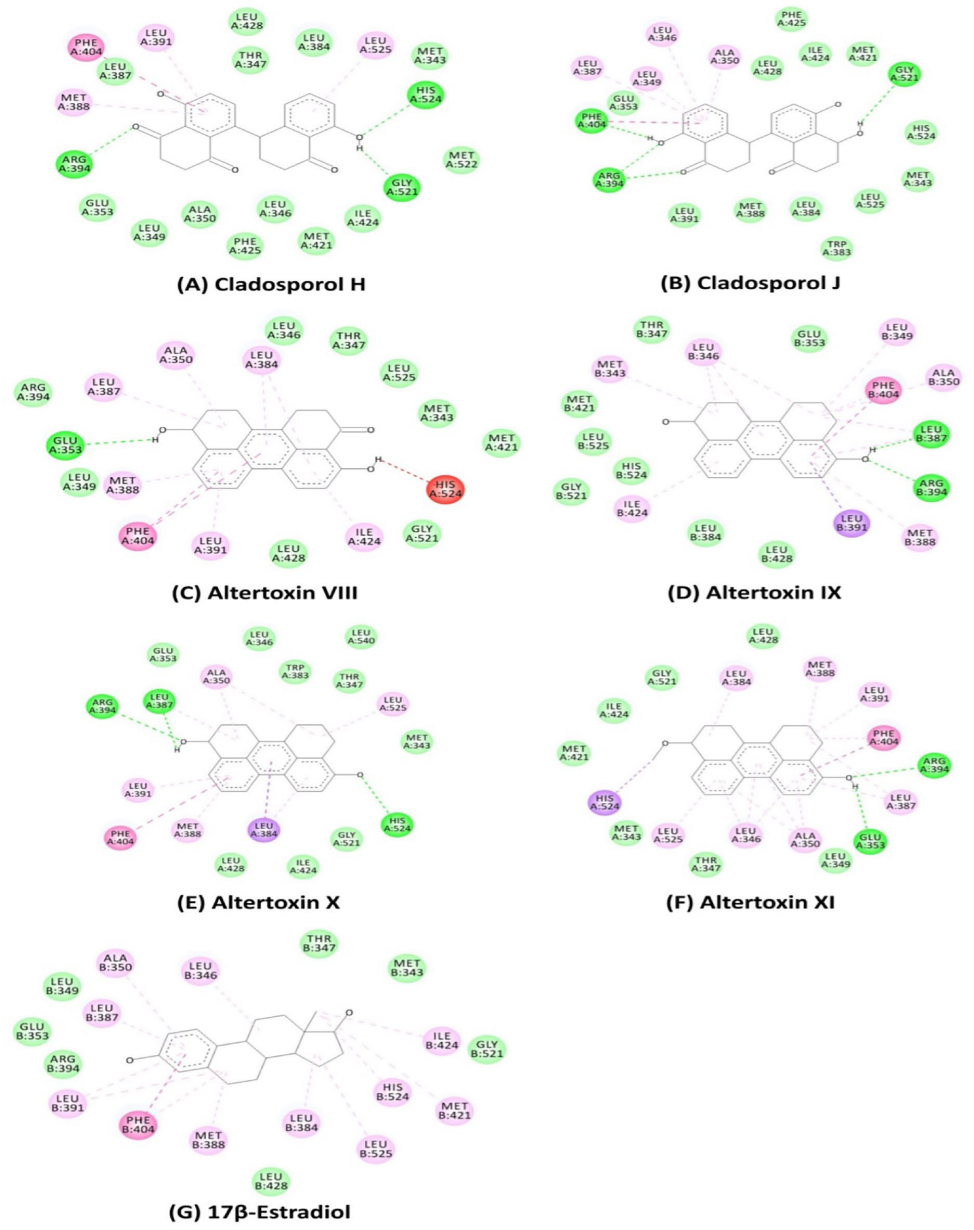
Non-bonding interaction of the potential bioactive compounds extracted from *Cladosporium* spp. against estrogen receptor alpha (PDB: 6CBZ) protein.

Besides, the hydrogen bonding significantly enhanced the binding energy and binding mode, which is important for influencing the ligand binding specificity with the receptor, drug design in chemical and biological processes, molecular recognition, and biological activity^42^. The best docked complex of Altertoxin X with the estrogen receptor alpha protein with binding energy − 10.5 kcal/mol was firmly bound through conventional hydrogen bonds with the residues of LEU387, ARG394 and HIS524 (Fig. 2). It has been reported that the molecular docking was utilized for virtual screening of the compounds produced in fungal endophytes (*Chaetomium* sp.) against the human estrogen receptor alpha (PDB: 1G50) for searching anti-breast cancer agents^43^. The observed binding energies for virtual active compounds ranged from − 9.2 to -4.9 kcal/mol with the corresponding active amino acid residues (such as ARG394, GLU323, GLU353, LEU345, LEU346, LYS449, PRO324, PRO325 and TRP393).

The findings of Ervina et al^44^ have noticed that Quercetin 3-O-(2”,6”-digalloyl)-$\upbeta$-D-galactopyranoside isolated from *Melia azearach* leaves bioactive ethyl acetate fraction offered binding energy of − 9.9 kcal/mol during molecular docking with estrogen receptor (PDB: 3ERT) which was lower that its agonist 17$\upbeta$-Estradiol (− 9.4 kcal/mol). The better potency of Quercetin 3-O-(2”,6”-digalloyl)-$\upbeta$-D-galactopyranoside to bind the estrogen receptor alpha indicatred its higher binding affinity to the active sites of 3ERT. In addition, Muhammad et al^45^ have reported that among the 10 selective bioactive Curcumin derivatives, Salicydenecurcumin, 4-benzylidene Curcumin, and difluorinated Curcumin were found to show the lowest binding energy of -8.6 kcal/mol, -8.8 kcal/mol, and − 9.0 kcal/mol, respectively with the same protein 3ERT. Therefore, the MD simulation studies were conducted with Altertoxin X and Cladosporol H based on their better binding potential along with the reference compound.

**Table 1:** Molecular docking results of the bioactive compounds extracted from *Cladosporium* spp. against estrogen receptor alpha (PDB: 6CBZ) protein.

SI No	Compound name	Binding energy (Kcal/mol)
1	Aspispermidin-20-ol 1-acetyl-17-methoxy	− 7.4
2	Cladosporine A	− 8.1
3	Cytochalasin D	− 8.0
4	2-Methylacetate-3,5,6-trimethylpyrazine	− 6.6
5	Lunatoic acid	− 7.0
6	(6bS,7R,8S)-4,9-Dihydroxi-7,8-dimethoxy-1,6b,7,8-tetra-hydro-2H-benzo[J]-	
	-fluoranthen-3-one	− 7.6
7	(6bR,7R,8S)-Methoxy-4,8,9-trihydroxy-1,6b,7,8-tetrahydro-2H-benzo[J]-	
	-fluoranthen-3-one	− 9.1
8	(6bS,7R,8S)-Methoxy-4,8,9-trihydroxy-1,6b,7,8-tetrahydro-2H-benzo[J]-	
	-fluoranthen-3-one	− 7.4
9	Coniochaetone A	− 8.1
10	Coniochaetone B	− 8.2
11	Coniochaetone K	− 8.5
12	Cladosporinone	− 7.3
13	Viriditoxin	− 7.5
14	Viriditoxin SC-28763	− 7.4
15	Viriditoxin SC-30532	− 7.6
16	Cladospolide F	− 6.7
17	Cladospolide G	− 6.9
18	Ent-Cladospolide F	− 6.6
19	11-Hydroy-gamma-dodecalactone	− 6.4
20	Iso-Cladospolide B	− 6.9
21	Citrinin H1	− 7.4
22	Cladosporin A	− 6.9
23	Cladosporin B	− 6.8
24	Cladosporin C	− 8.3
25	Cladosporin D	− 6.7
26	Cladosporin	− 9.2
27	5’-Hydroxyasperentin	− 9.4
28	Isocladosporin	− 8.6
29	Cladoscyclitol B	− 6.7
30	3-Hydroxy-2,4,5-trimethylphenyl-4-[(2,4-dihydroxy-3,6-dimethylbenzoyl)oxy]-	
	2-hydroxy-3,6-dimethylbenzoate	− 8.5
31	3-Hydroxy-2,5-dimethylphenyl-2,4-dihydroxy-3,6-dimethylbenzoate	− 9.3
32	3-Hydroxy-2,4,5-dimethylphenyl-4-[(2,4-dihydroxy-3,6-dimethylbenzoyl)oxy]-	
	2-hydroxy-3,6-dimethylbenzoate	− 7.7
33	(2S)-7,4’-Dihydroxy-5-methoxy-8-(gamma,gamma-dimethyallyl(-favanone	− 7.3
34	Cladosporamide A	− 8.6
35	Cladocladosin A	− 7.9
36	Cladospolide B	− 7.9
37	5R-Hydroxyrecifeiolide	− 7.4
38	5S-Hydroxyrecifeiolide	− 7.9
39	5Z-7-Oxozeaenol	− 7.7
40	Pandangolide 1	− 7.4
41	Pandangolide 3	− 7.1
42	Sporiolide A	− 8.5
43	Sporiolide B	− 7.9
44	Thiocladospolide A	− 8.0
45	Thiocladospolide B	− 7.8
46	Thiocladospolide C	− 7.5
47	Thiocladospolide D	− 7.0
48	Thiocladospolide F	− 8.0
49	Thiocladospolide F bis	− 8.7
50	Thicladospolide G	− 7.9
51	Thicladospolide G bis	− 7.7
52	Thicladospolide H	− 8.3
53	Thicladospolide I	− 7.3
54	Thicladospolide J	− 7.7
55	Zeanol	− 8.0
56	Cladonaphehrom A	− 7.4
57	Cladonaphehrom B	− 6.9
58	Cladosporol A	− 8.7
59	Cladosporol B	− 8.1
60	Cladosporol C	− 9.6
61	Cladosporol D	− 8.0
62	Cladosporol E	− 8.2
63	Cladosporol F	− 8.6
64	Cladosporol G	− 8.1
65	Cladosporol G bis	− 7.4
66	Cladosporol H	− 10.3
67	Cladosporol I	− 8.5
68	Cladosporol J	− 9.9
69	Cladosporone A	− 7.7
70	(3S)-3,8-Dihydroxy-6,7-dimethyl-alpha-tetralone	− 7.7
71	Scytalone	− 7.2
72	Anhydrofusarubin	− 7.5
73	Fusarubin methyl ether	− 7.2
74	Altertoxin VIII	− 9.9
75	Altertoxin IX	− 9.9
76	Altertoxin X	− 10.5
77	Altertoxin XI	− 9.9
78	Altertoxin XII	− 9.4
79	Calphostin A	− 6.2
80	Calphostin B	− 6.5
81	Calphostin C	− 7.3
82	Calphostin D	− 6.9
83	Calphostin I	− 9.2
84	Pheichrome	− 6.1
85	Cladospolide E	− 6.5
86	Seco-patulolide A	− 6.5
87	Seco-patulolide C	− 6.2
88	(3S,5S,11S)-Trihydroxydodecanoic acid	− 6.3
89	Cladosporide A	− 7.1
90	Cladosporide B	− 7.3
91	Cladosporide C	− 6.9
92	3-alpha-Hydroxy-pregn-7-ene-6,20-dione	− 9.0
93	Cladodionen	− 8.0
94	Cladosin B	− 6.9
95	Cladosin C	− 7.3
96	Cladosin F	− 7.1
97	Cladosin I	− 7.0
98	Cladosin J	− 7.6
99	Cladosin K	− 7.3
100	Cladosin L	− 7.2
101	Cladosin L bis	− 7.3
102	Cladosporicin A	− 7.8
103	Cladosporiumin I bis	− 8.6
104	Cladosporiumin J bis	− 6.8
105	Malettinin A	− 7.6
106	Malettinin B	− 8.6
107	Malettinin C	− 9.5
108	Malettinin E	− 8.3
109	Conioxanthone A	− 7.5
110	3,8-dihydroxy-6-methyl-9-oxo-9H-xanthene-1-carboxylate	− 7.1
111	Alpha-Diversonolic ester	− 8.3
112	Beta-Diversonolic ester	− 8.9
113	8-Hydroxy-6-methylxanthone-1-carboxylic acid	− 8.7
114	Methyl-8-hydroxy-6-(hydroxymethyl)-9-oxo-9H-xanthene-1-carboxylate	− 8.5
115	Methyl-8-hydroxy-6-methyl-9-oxo-9H-xanthene-1-carboxylate	− 8.5
116	8-(Methoxycarbonyl)-1-hydroxy-9-oxo-9H-xanthene-3-carboxylic acid	− 9.0
117	Vertixanthone	− 8.2
118	Acetyl Sumiki’s acid	− 6.3
119	1,1’-Dioxine-2,2’-dipropionic acid	− 6.6
120	4-O-alpha-D-ribofuranose-2-pentyl-3-phemethylol	− 6.7
121	Sumiki’s acid	− 5.6
122	Taxol	− 7.1
123	Vermistatin	− 7.0
124	17$\upbeta$-Estradiol (Reference Compound)	− 10.2

### Protonation states of 6CBZ protein and selected ligands

Among the different amino acids, only His amino acid residues are known to play a physiological role at neutral pH due to different protonated structures based on the pKa value around them^46^. At neutral pH, the His residues in proteins can attain three different protonation states: (1) Hid protonation has a hydrogen atom at the $\updelta$-site of its imidazole ring; (2) Hie protonation has a hydrogen atom at the $\upepsilon$-site of the imidazole ring, and (3) positively charged Hip protonation has hydrogen atoms at both the $\updelta$- and $\upepsilon$-sites. Therefore, the His residues with pKa value > 6 have the Hip protonation, while those residues with pKa value <6 have the Hid or Hie protonation^18^ . During the determination of the protonation state of the protein selected (6CBZ), it was noted that the protein consists of 24 His residues with varying pKa values (Supplementary Table 1) wherein it was noted that only 12 His residues had pKa values < 6 and designated to have Hie protonation. The His residues with only Hie protonation were considered because practically it is impossible to consider all the combinations of Hid/Hie protonation.

In addition, the protein binding potential of the molecules can be encouraged or discouraged by changing hydrogen-bond, electrostatic, and Van der Waals interactions. During protonation, the proton (or hydrogen cation, H$^+$) is added to an atom, molecule, or ion to form a conjugate acid, thereby enhancing its binding affinity with the protein receptor. The study results showed that Altertoxin X, Cladosporol H and 17$\upbeta$-Estradiol at the neutral pH were found to be in protonated state (pKa > 7), thereby improving their binding ability.

### Molecular dynamics simulation studies

**Figure 3:**
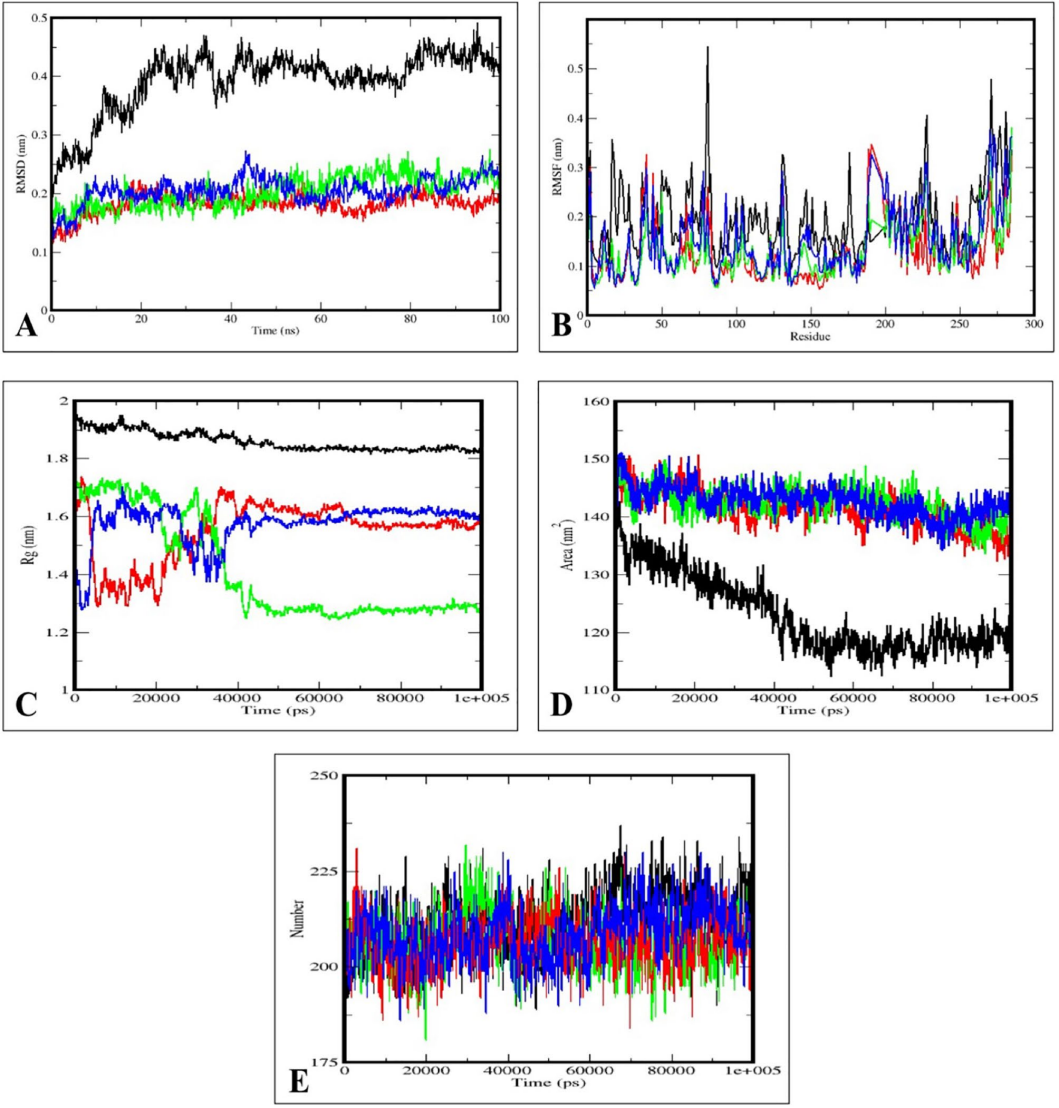
The molecular dynamics simulation trajectories from 100 ns simulation time represents the (**A**) RMSD plot, (**B**) RMSF plot, (**C**) RG plot, (**D**) SASA plot, and (**E**) Hydrogen bond plot (Black: 6CBZ alone, Red: 6CBZ-Altertoxin X complex, Blue: 6CBZ-Cladosporol H complex, and Green: 6CBZ-17$\upbeta$-Estradiol complex.

The best docked bioactive compounds, Altertoxin X and Cladosporol H, along with a reference compound (17$\upbeta$-Estradiol) in complex with the receptor (estrogen receptor alpha; PDB: 6CBZ), were further subjected to the molecular dynamics simulation to study the interaction and stability of the protein-ligand complex in an aqueous system for a simulation time of 100 ns. The simulation study provided the geometric properties of the protein-ligand complex that included the analysis of RMSD (Root Mean Square Deviation), RMSF (Root Mean Square Fluctuation), Rg (Radius of Gyration), SASA (Solvent Accessible Surface Area) and a number of hydrogen bonds maintained throughout the simulation time and variation in receptor alone and the three complexes (Figure 3)^47^. These calculations were analyzed to understand the simulation systems’ structural variations and stability. The RMSD of atomic positions is used to calculate the average distance between the atoms of superimposed protein and ligand structures over time^48^. The RMSD plot of the estrogen receptor alpha protein alone showed that the estrogen receptor alpha protein alone reached the equilibrium approximately at 80–100 ns time and the remaining showed the stable trajectory simulation with minimal deviation in 0.35-0.47 nm RMSD range, whereas the RMSD plot of estrogen receptor alpha-Altertoxin X complex reached the equilibrium at 40–100 ns and 0.15-0.21 nm. The structural flexibility of protein is reserved when the estrogen receptor alpha protein is in free form in its complex with Altertoxin X compared to Cladosporol H and 17$\upbeta$-Estradiol. The ligand molecule, Altertoxin X bound to the estrogen receptor alpha protein, reached equilibrium after initial fluctuations, showing that this molecule has fewer deviations than the other molecules.

The RMSF concentrates on the protein structural regions that differ the most/least from the mean. Further, by calculating root mean square distances with respect to the central axis of rotation. The RMSF plots were predicted with fluctuations only at the terminal ends and loop regions, indicating the stable interactions between the complexes. The protein complexed with Altertoxin X exhibited fewer fluctuations, indicating it to be a stable molecule. The Rg is the root mean square distance between each protein atom and its center of mass in a system^49^ . The Rg plot analyzed the capability, shape and folding during every time step of the trajectory throughout the simulation. The estrogen receptor alpha protein complexed with Altertoxin X exhibited a similar pattern of Rg values with a deviation of 1.3–1.7 nm. The solvent-accessible surface area of the simulation complex was explored to understand better the complex’s changes in surface area, where a higher SASA indicates the extension of surface volumes and a lower SASA indicates the truncated nature of the complex^50^. The SASA calculates the surface area of the hydrophobic core generated by protein-ligand interactions. Consistent SASA values were observed in the estrogen receptor alpha protein-Altertoxin X complex compared to other complexes.

In addition, the hydrogen bond must be assessed in the biological system to determine the bonding and structural changes in the complex. The stability of the complex is defined by the hydrogen bond of the simulation system, where the entire complex exhibits a stable trend^51^. The hydrogen bonds during molecular docking are evaluated across the simulation duration. All intermolecular hydrogen bonds between estrogen receptor alpha (protein) and Altertoxin X (ligand) were solely examined during the analysis and shown accordingly. The plot showed that the number of hydrogen bonds formed during simulation runs was consistent with the molecular docking study, with only a few bonds being broken and repaired simultaneously compared to Cladosporol H and 17$\upbeta$-Estradiol. The MD simulation verified the stability of docked complexes Salicylidenecurcumin and Curcumin difluorinated within the interaction cavity of the estrogen receptor alpha 3ERT in humans and confirmed the steadiness of the complexes over the simulated trajectories at 120 ns time scale^45^.

### Binding free energy calculations

Various energy metrics such as Van der Waals, electrostatic, polar solvation, SASA, and binding energies were utilized to measure the extent of ligand-target protein binding interactions during MD simulations. In this study, the protein-ligand complex was majorly formed using the Van der Waals energy followed by the binding energy, SASA energy, and electrostatic energy. Polar solvation energy was predicted with no contribution to the protein-ligand complex formation, as the values appeared positive. The 6CBZ complexed with Altertoxin X, Cladosporol H, and 17$\upbeta$-Estradiol (reference compound) were considered for binding energy calculation studies. In addition, the protein-ligand complex standard deviations were calculated. A lower standard deviation means the data values are closer to the mean (or expected value), whereas a high standard deviation means the data values are spread out over a wider range. However, there was no high standard deviation in the Altertoxin X- receptor complex compared to Cladosporol H and 17$\upbeta$-Estradiol, which has high standard deviation values, which indicates that Altertoxin X binds to the protein with high binding affinity and stable interaction. The binding free energy calculations of the protein-ligand complex have been represented in Table 2:

**Table 2:** Binding free energy calculations of 6CBZ target protein complexes with Altertoxin X, Cladosporol H, and 17$\upbeta$-Estradiol (all results in kJ/mol).

Categories	6CBZ-Altertoxin X complex		6CBZ-Cladosporol H complex		6CBZ-17$\upbeta$-Estradiol complex	
	Values	Standard deviation	Values	Standard deviation	Values	Standard deviation
Van der Waals energy	− 189.746	±159.137	− 224.439	±195.457	− 267.875	± 214.784
Electrostatic energy	− 45.672	± 39.198	− 32.576	± 15.198	− 67.457	± 24.918
Polar solvation energy	78.164	± 68.852	96.768	± 46.832	98.446	± 64.018
SASA energy	− 23.120	± 15.227	18.891	± 13.677	42.872	± 38.425
Binding energy	145.440	±129.163	− 193.378	±166.163	− 231.232	± 187.124

### Absorption, distribution, metabolism, excretion and toxicity (ADMET) prediction

Oral bioavailability is one of the most important properties in drug design. A higher score reduces the amount of an administered drug necessary to achieve the desired pharmacological effect, thereby lessening the risk of side effects and toxicity^52^. The 90% of orally bioactive drugs that have progressed to the second stage of the clinical trial are associated with the four simple physicochemical properties ranges the molecular weight $\le$ 500, logP $\le$ 5, number of hydrogen bond acceptors $\le$ 10, and number of hydrogen bond donors $\le$ 5^53^. The excellent physicochemical properties of Altertoxin X (Table 3) are responsible for its interaction with the amino acid residues of estrogen receptor alpha protein by forming hydrogen bonds. In addition, the orally bioactive drugs that are passively transported via the transcellular route should not exceed their polar surface area of about 120 Å^2^ and should be tailored to less than 60–70 Å^2^ for good brain penetration of drugs^54^. The polar surface area $\le$140 Å^2^ predicted to have a high probability of good oral bioavailability. Altertoxin X was found to have good oral bioavailability. The prediction of ADMET properties is critical in drug discovery and development because these properties are responsible for around 60% of all drug failures in clinical trials. The ADMET is used early in the drug development process to eliminate compounds with poor ADMET properties from the pipeline, reducing the research and development cost. In addition, the ADMET analysis of the selected compounds corroborates with the MD simulation studies’ findings, thereby supporting the utilization of the same for further in vitro and in vivo studies (Table 4 and Supplementary Table 2).

The absorption of drugs from an orally administered solution depends on the factors including water solubility, Caco-2 cell membrane permeability, human intestinal absorption, skin permeability threshold, and substrate or inhibitor of P-glycoprotein. High water solubility is one of the useful factors for delivering a sufficient quantity of active ingredients in the small volume of such pharmaceutical dosage^55^. According to log S scale, the water solubility of the drug molecules is considered as poorly soluble if the value is < − 10 mol/L; moderately soluble if < − 6 mol/L; soluble if < − 4 mol/L; very soluble if < − 2 mol/L, and highly soluble if < 0 mol/L. From the results, it was observed that Altertoxin X tested was moderately soluble in water. The apparent permeability coefficient (Papp) value of drug molecules measures the rate at which they can cross the area of Caco-2 cell monolayer. The high Caco-2 permeability is translated into the predicted log Papp value of $\ge$ 0.90 cm/s in the pkCSM predictive model. The predicted Caco-2 permeability value (log Papp 1.612 cm/s) of Altertoxin X indicated a low Caco-2 permeability. The human intestinal absorption value greater than 90% for Altertoxin X indicated its excellent absorption. The skin permeability (Kp) measures the rate at which the drug molecules penetrate the stratum corneum. The Kp value is widely used to quantitatively describe the transport of drug molecules into the outermost layer of epidermal skin (or stratum corneum) and indicates the significance of skin absorption^39^. The drug molecules will easily penetrate the skin if their log Kp value is > − 2.5 cm/h. Altertoxin X also has the predicted log Kp value of − 2.738 cm/h ($\le$ − 2.5 cm/h) indicating its poor skin permeability. P-glycoprotein is a member of the ATP-binding cassette (ABC) transporter family that actively transports various compounds out of the cells. Here, Altertoxin X was predicted to be transported across the cell membrane through ABC transporter.

The steady-state volume of distribution (VDss) is one of the pharmacokinetic parameters that representing the propensity of drugs to either remain in blood plasma or redistribute to another tissues^56^. The high VDss value (> 0.45 L/kg) indicates the propensity of drugs to leave the plasma and enter the other tissues of the body, while the low VDss value (< − 0.15 L/kg) indicates the propensity of drugs to remain in the plasma. A higher drug dose is required to achieve in a given plasma concentration if it has a high VDss value due to more distribution of drug to other tissues. Conversely, a lower drug dose is required to achieve in a given plasma concentration if it has a low VDss value due to less drug distribution to other tissues. The compound Altertoxin X used in the present study has a relatively low VDss value (0.025 L/kg). The prediction of blood-brain barrier (BBB) permeability is an important factor for regulating the transportation of drugs from and to the central nervous system (CNS)^57^. The log BB is the most common numerical value describing the BBB permeability. In the qualitative model, the drug is considered BBB permeable if the log BB value $\ge$ 0.3 and non-permeable if the log BB value $\le$ − 0.3. Altertoxin X could be easily passed the BBB because it has the log BB value of 0.331. In addition, the drugs that have log PS value $\ge$ − 2 are considered to penetrate the CNS, while those with log PS value $\le$ − 3 have difficulty in penetrating the CNS. It can be noticed that Altertoxin X with the log PS value − 1.839 was believed to penetrate the CNS.

The inhibition of major human cytochrome P450 (CYPs) monooxygenase enzymes is involved in the metabolism of drugs in order to prevent undesirable adverse effects. It was observed that Altertoxin X was found to inhibit all the enzymes except CYP2D6 and CYP3A4; thus it could not be metabolized by the CYPs monooxygenase enzymes in the body. The clearance of drugs quantitatively describes the volume of plasma from which they would be removed per unit. The pkCSM pharmacokinetics model predicts the given compounds’ total clearance (log mL/min/kg). The higher total clearance value of the compounds indicates their faster excretion processes. The predicted excretion rate of Altertoxin X was 0.003 mL/min/kg. The positive AMES toxicity test suggests that Altertoxin X was mutagenic. Altertoxin X has high LD$_{50}$ value (2.375 mol/kg), which indicates that it was lethal to the human body only at extremely high dose. Moreover, it did not cause any hepatotoxicity and skin sensitization.

**Table 3:** Chemical structural properties of the selected ligands.

Descriptor	Altertoxin X	Cladosporol H	17$\upbeta$-Estradiol
Molecular Weight (g/mol)	290.36	336.343	272.388
LogP	4.16	3.3654	3.6092
Rotatable Bonds	0	1	0
Hydrogen Bond Acceptors	2	5	2
Hydrogen Bond Donors	2	2	2
Polar Surface Area (Å^2^ )	128.56	143.469	120.382

**Table 4:** Predicted ADMET properties of the selected ligands.

Descriptor	Model Name	Altertoxin X	Cladosporol H	17$\upbeta$-Estradiol
Absorption	Water solubility (log mol/L)	− 5.463	− 3.442	− 3.803
	Caco2 permeability (log Papp in 10$^{-6}$ cm/s	1.612	0.794	1.766
	Intestinal absorption (human) (% Absorbed	95.815	100	93.898
	Skin Permeability	− 2.738	− 2.753	2.970
	P-glycoprotein substrate	Yes	Yes	Yes
	P-glycoprotein I inhibitor	No	No	No
	P-glycoprotein II inhibitor	Yes	No	No
Distribution	VDss (human) (log L/kg)	0.025	0.069	0.549
	Fraction unbound (human) (Fu)	0.007	0.054	0.111
	BBB permeability	0.331	0.029	− 0.072
	CNC permeability (log PS)	− 1.839	− 2.067	− 1.330
Metabolism	CYP2D6 substrate	No	No	No
	CYP3A4 substrate	Yes	Yes	Yes
	CYP1A2 inhibitor	Yes	Yes	Yes
	CYP2C19 inhibitor	Yes	Yes	Yes
	CYP2C9 inhibitor	Yes	Yes	Yes
	CYP2D6 inhibitor	No	No	No
	CYP3A4 inhibitor	No	Yes	No
Excretion	Total Clearance (log ml/min/kg)	0.003	0.148	0.784
	Renal OCT2 substrate	No	No	No
Toxicity	AMES toxicity	Yes	No	No
	Max. tolerated dose (human) (log mg/kg/day)	0.521	− 0.544	− 0.677
	hERG I inhibitor	No	No	No
	hERG II inhibitor	Yes	No	Yes
	Oral Rat Acute Toxicity (LD50) (mol/kg)	2.375	2.321	2.697
	Oral Rat Chronic Toxicity (LOAEL) (log mg/kg$\_$bw/day)	1.610	1.847	1.993
	Hepatotoxicity	No	No	No
	Skin Sensitization	No	No	No
	*T. pyriformis* (log $\upmu$g/L)	0.391	0.356	1.163
	Minnow toxicity (log mM)	− 0.589	0.904	0.541

Caco-2, Colon cancer cell line; Papp, apparent permeability coefficient; Kp, skin permeability constant; VDss, volume of distribution at steady state; Fu, fraction unbound; BBB, blood-brain-barrier; CBS, central nervous system; PS, permeability-surface area; CYP, Cytochrome P450; AMES, assay of the compounds ability to induce mutations in DNA; hERG, human ether-a-go-ago related gene; LD, lethal dose; LOAEL, lowest observed adverse effect level; *T. pyriformis , Tetrahymena pyriformis*.

### Conceptual DFT studies

Conceptual Density Functional Theory (CDFT) is the branch of DFT that deals with the chemical reactivity of atoms and molecules and their interacting behavior. As such, it offers a large number of tools for the study the interactions between chemical systems. Thus, it has great importance in the process of drug design and discovery, mainly through virtual screening. It is expressed in the form of descriptors that can help to get a glimpse of how drugs interact with the receptors in the body. Indeed, this is not an easy task because those descriptors cannot be always related to the physiological chemistry of the human body. However, the estimation of the CDFT descriptors can help to get a qualitative idea of how these interactions proceed.

The structures of the selected ligands, Altertoxin X and Cladosporol H, together with the reference compound, 17$\upbeta$-Estradiol, have been optimized by following the methodology presented in the Materials and Methods section. We have resorted to three different functionals: B3LYP^58–60^, PBE0^61^ and MN12SX^31^, being in all cases, Def2TZVP^32,33^ the chosen basis set, and ${\hbox {H}_{2}\hbox {O}}$ the solvent simulated with the SMD model^34^. The required frequencies calculations for the verification of the absence of imaginary frequencies as a check for the stability of the optimized structures as being a minimum in the energy landscape was performed for all the systems.

The quality of the chosen density functionals may be realized by comparing their results with those from high-level computations or from experimental values. Nevertheless, this comparison is not always computationally practicable because of the large size of the molecules or the lack of experimental results for the chemical methods being explored. Our research group has developed a methodology known as KID^35–38^, in order to evaluate a particular density functional with regard to its internal coherence. Within the Generalized Kohn-Sham (GKS) version of DFT, some relationships exist between the KID methodology and the Ionization Energy Theorem, which is a corollary of Janak theorem^62,63^. This is done by connecting $\epsilon _H$ to -I and $\epsilon _L$ to -A, through $J_{I} = {\epsilon _H + E_{gs}(N-1) - E_{gs}(N)}$, $J_{A} = \epsilon _L + E_{gs}(N) - E_{gs}(N+1)$ and $J_{HL} = \sqrt{{J_{I}}^2 + {J_{A}}^2}$, being $\epsilon _H$ and $\epsilon _L$ the frontier orbital energies of the studied molecules. Another KID descriptor $\Delta$SL related to the difference in energies between the SOMO and the LUMO of the neutral system has been devised to aid in the verification of the accuracy of the methodology. A general Global KID Descriptor has been defined as ${\mathrm{GKD}} = \sqrt{{J_{I}}^2 + {J_{A}}^2 + {J_{HL}}^2 + {\Delta {SL}}^2}$, whose value must be zero for the exact density functional meaning that it verifies the Ionization Energy theorem. The results are presented in Table 5:

**Table 5:** Average GDK values for Altertoxin X, Cladosporol H, and 17$\upbeta$-Estradiol considering three density functionals (B3LYP, PBEO and MN12SX) (all results in eV).

Ligand	B3LYP	PBE0	MN12SX
Altertoxin X	0.6713	1.0911	0.0201
Cladosporol H	0.6030	1.0659	0.0277
17$\upbeta$-Estradiol	0.7288	1.2757	0.0591

It can be concluded from an evaluation of the results in Table 5 that the MN12SX density functional is the best for verifying the Ionization Energy theorem while the B3LYP and PBE0 density functionals will render values of the calculated properties with large errors. Thus, the calculated results for the Global Reactivity Descriptors derived from CDFT (including the Nucleophilicity N)^21–24^ for the Altertoxin X, Cladosporol H, and 17$\upbeta$-Estradiol acquired utilizing the in-house CDFT tool software based on the MN12SX/Def2TZVP/H2O geometry optimizations and frequency calculations are displayed in Table 6:

**Table 6:** Global reactivity descriptors or Altertoxin X, Cladosporol H, and 17$\upbeta$-estradiol considering the MN12SX/Def2TZVP/H2O model chemistry.

Ligand	$\chi$	$\eta$	$\omega$	S	N	$\omega ^{-}$	$\omega ^{+}$	$\Delta \omega \pm$
Altertoxin X	3.6243	4.0986	1.6024	0.2440	3.1189	5.2732	1.6489	6.9221
Cladosporol H	4.5640	3.5715	2.9162	0.2800	2.4427	8.3376	3.7736	12.1112
17$\upbeta$-Estradiol	3.3115	5.3664	1.0217	0.1863	2.7978	4.0346	0.7231	4.7577

Note: $\chi$ - Electronegativity; $\eta$ - Global Hardness; $\omega$ - Electrophilicity; S - Global Softness; N - Nucleophilicity; $\omega ^{-}$ - Electrodonating Power; $\omega ^{+}$ - Electroaccepting Power; $\Delta \omega \pm$ - Net Electrophilicity. All the descriptors are expressed in eV, with the exception of S, which is expressed in eV$^{-1}$.

The electronegativity $\chi$ and global hardness $\eta$ are absolute chemical reactivity parameters for which no experimental analogue exists. Indeed, the observed vertical ionization energy (I) and vertical electron affinity (A) can be used to approximate them, but these values are unknown for the molecular system under investigation. However, in comparing Altertoxin X and Cladosporol H and the results from Table 1, it can be said that they correlate with their global hardness values. The electrophilicity index $\omega$ is a balance between an electrophile’s proclivity for acquiring more electron density and its reluctance to exchanging electron density with its surroundings^64^. An electrophilicity scale was established by Domingo et al^65–67^, with larger than 1.5 eV for strong electrophiles, between 0.8 and 1.5 eV for medium electrophiles, and smaller than 0.8 eV for the marginal ones^65–67^, On the basis of Table 5, Altertoxin X and Cladosporol H may be classified as strong electrophiles while 17$\upbeta$-Estradiol is a medium electrophile. In the same way, Domingo and colleagues^68,69^ presented a Nucleophilicity index N based on the HOMO energy calculated using the KS technique with an arbitrary shift of the origin, using the molecule of tetracyanoethylene (TCE) as a reference. They were able to classify organic molecules as strong nucleophiles with N > 3.0 eV, moderate nucleophiles with 2.0 < N < 3.0 eV, and marginal nucleophiles with N < 2.0 eV. By re-examining Table 6, it is clear that Altrotoxin X may be classified as a strong nucleophile while Cladosporol H will act as a moderate nucleophile. Although the difference between the binding energies for Altrotoxin X and Cladosporol H is small, perhaps the better behavior of the first could be attributed to its observed nucleophilicity. The same conclusions may obtained by relating the binding energies to the inverse of the $\omega ^{-}$, $\omega ^{+}$ and $\Delta \omega \pm$. These results could also be considered as complement of a recent study^70^, where Altertoxin X was identified as new perylenequinone found in the marine derived fungus *Cladosporium* spp. possessing quorum sensing inhibitory activity against *Chromobacterium violaceum* with a minimum inhibitory concentration (MIC) of 20 $\upmu$g/well, and whose structure was elucidated through quantum ECD calculations.

## Conclusion

Since Alexander Fleming discovered Penicillin, the microbes have also been considered a rich source of most bioactive compounds. In the present study, the integrated approaches of Molecular Docking and MD simulation studies, as well as DFT calculations, were utilized to explore the structural insights into possible binding modes of drug-like bioactive compounds of *Cladosporium* spp. against the estrogen receptor alpha. Among the compounds screened, Altertoxin X and Cladosporol H were found to have better binding affinity than the reference standard (17$\upbeta$-Estradiol). The MD simulation study for up to 100 ns confirmed that the complexes of estrogen receptor alpha with Altertoxin X and Cladosporol H were highly stable in the biological system. The ADMET results revealed that Altertoxin X has a good oral bioavailability with a high LD$_{50}$ value (2.375 mol/kg) and did not cause hepatotoxicity or skin sensitization. From the present study, it can be concluded that Altertoxin X and Cladosporol H, which possess the most significant inhibitory potential against the estrogen receptor alpha than the reference compound (17$\upbeta$-Estradiol), were reported as the antagonists of estrogen receptor alpha and as the promising therapeutic drug candidates for the treatment of breast cancer. Therefore, they can be considered candidates for pre-clinical and clinical trials as the future of clinical therapy. In a future study, the in vitro and in vivo studies will also be conducted to further validate the potential of Altertoxin X and Cladosporol H as clinical drugs for breast cancer treatment before the clinical trial.

## Supplementary Information

Supplementary Tables.

## Data Availability

The datasets generated during and/or analyzed during the current study are available from the corresponding author on reasonable request.
