# Author Correction: Integrated approach for studying bioactive compounds from *Cladosporium* spp. against estrogen receptor alpha as breast cancer drug target

**DOI:** 10.1038/s41598-023-28772-0

**Published:** 2023-02-09

**Authors:** Satish Anandan, Hittanahallikoppal Gajendramurthy Gowtham, C. S. Shivakumara, Anjana Thampy, Sudarshana Brijesh Singh, Mahadevamurthy Murali, Chandan Shivamallu, Sushma Pradeep, Natarajamurthy Shilpa, Ali A. Shati, Mohammad Y. Alfaifi, Serag Eldin I. Elbehairi, Joaquín Ortega‑Castro, Juan Frau, Norma Flores‑Holguín, Shiva Prasad Kollur, Daniel Glossman‑Mitnik

**Affiliations:** 1 Department of Clinical Nutrition and Dietetics, Sri Devaraj Urs Academy of Higher Education and Research, Kolar, Karnataka 563101 India; 2 Department of PG Studies in Biotechnology, Government Science College (Autonomous), Nrupathunga Road, Bangalore, 560001 India; 3 Department of Studies in Botany, University of Mysore, Manasagangotri, Mysore, Karnataka 570006 India; 4 Department of Biotechnology and Bioinformatics, JSS Academy of Higher Education and Research, Mysore, Karnataka 570015 India; 5 Department of Studies in Microbiology, University of Mysore, Manasagangotri, Mysore, Karnataka 570006 India; 6 Biology Department, Faculty of Sciences, King Khalid University, Abha, Saudi Arabia; 7 Cell Culture Lab, Egyptian Organization for Biological Products and Vaccines (VACSERA Holding Company), 51 Wezaret El‑Zeera St., Agouza, Giza, Egypt; 8 Departament de Química, Universitat de les Illes Balears, 07122 Palma de Mallorca, Spain; 9 Laboratorio Virtual NANOCOSMOS, Departamento de Medio Ambiente y Energía, Centro de Investigación en Materiales Avanzados, 31136 Chihuahua, Chih, Mexico; 10 School of Physical Sciences, Amrita Vishwa Vidyapeetham, Mysuru Campus, Mysuru, Karnataka 570 026 India

Correction to: *Scientific Reports* https://doi.org/10.1038/s41598-022-22038-x, published online 27 December 2022

The original version of the Article contained an error in the Author Information section.

“These authors contributed equally: Satish Anandan, Hittanahallikoppal Gajendramurthy Gowtham, C. S. Shivakumara, Anjana Thampy, Sudarshana Brijesh Singh, Mahadevamurthy Murali, Chandan Shivamallu, Sushma Pradeep, Natarajamurthy Shilpa, Ali A. Shati, Mohammad Y. Alfaifi, Serag Eldin I. Elbehairi, Joaquín Ortega‑Castro, Juan Frau, Norma Flores‑Holguín, Shiva Prasad Kollur and Daniel Glossman‑Mitnik.”

now reads:

“These authors contributed equally: Satish Anandan and Hittanahallikoppal Gajendramurthy Gowtham.”

In addition, Shiva Prasad Kollur was incorrectly affiliated with “Department of Clinical Sciences, College of Veterinary Medicine, Kansas State University, Manhattan, Kansas 66506‑5606, USA” and “Midwest Veterinary Services, Inc., Oakland, NE 68045, USA”.

The correct affiliation is listed below.

School of Physical Sciences, Amrita Vishwa Vidyapeetham, Mysuru Campus, Mysuru, Karnataka, 570 026, India.

The author, Mohammad Y. Alfaifi, was incorrectly affiliated with “Cell Culture Lab, Egyptian Organization for Biological Products and Vaccines (VACSERA Holding Company), 51 Wezaret El-Zeera St., Agouza, Giza, Egypt”.

The correct affiliation is listed below:

Biology Department, Faculty of Sciences, King Khalid University, Abha, Saudi Arabia.

The original version of this Article also contained an error in Affiliation 10, which was incorrectly given as ‘Department of Clinical Sciences, College of Veterinary Medicine, Kansas State University, Manhattan, Kansas 66506‑5606, USA.’ The correct affiliation is listed below:

School of Physical Sciences, Amrita Vishwa Vidyapeetham, Mysuru Campus, Mysuru, Karnataka, 570 026, India.

In addition, Affiliation 11 ‘Midwest Veterinary Services, Inc., Oakland, NE 68045, USA’ was removed.

The original Article has been corrected.

